# The Fungal Gut Microbiome Exhibits Reduced Diversity and Increased Relative Abundance of Ascomycota in Severe COVID-19 Illness and Distinct Interconnected Communities in SARS-CoV-2 Positive Patients

**DOI:** 10.3389/fcimb.2022.848650

**Published:** 2022-04-19

**Authors:** Johanna Reinold, Farnoush Farahpour, Ann-Kathrin Schoerding, Christian Fehring, Sebastian Dolff, Margarethe Konik, Johannes Korth, Lukas van Baal, Jan Buer, Oliver Witzke, Astrid M. Westendorf, Jan Kehrmann

**Affiliations:** ^1^ Department of Infectious Diseases, University Hospital Essen, University of Duisburg-Essen, Essen, Germany; ^2^ Bioinformatics and Computational Biophysics, University of Duisburg-Essen, Essen, Germany; ^3^ Institute of Medical Microbiology, University Hospital Essen, University of Duisburg-Essen, Essen, Germany; ^4^ Department of Nephrology, University Hospital Essen, University of Duisburg-Essen, Essen, Germany; ^5^ Department of Endocrinology, Diabetes and Metabolism, University Hospital Essen, University Duisburg-Essen, Essen, Germany

**Keywords:** mycobiome, SARS-CoV-2, COVID-19, intestinal microbiota, co-occurrence network, severity

## Abstract

Clinical and experimental studies indicate that the bacterial and fungal gut microbiota modulates immune responses in distant organs including the lungs. Immune dysregulation is associated with severe SARS-CoV-2 infection, and several groups have observed gut bacterial dysbiosis in SARS-CoV-2 infected patients, while the fungal gut microbiota remains poorly defined in these patients. We analyzed the fungal gut microbiome from rectal swabs taken prior to anti-infective treatment in 30 SARS-CoV-2 positive (21 non-severe COVID-19 and 9 developing severe/critical COVID-19 patients) and 23 SARS-CoV-2 negative patients by ITS2-sequencing. Pronounced but distinct interconnected fungal communities distinguished SARS-CoV-2 positive and negative patients. Fungal gut microbiota in severe/critical COVID-19 illness was characterized by a reduced diversity, richness and evenness and by an increase of the relative abundance of the Ascomycota phylum compared with non-severe COVID-19 illness. A dominance of a single fungal species with a relative abundance of >75% was a frequent feature in severe/critical COVID-19. The dominating fungal species were highly variable between patients even within the groups. Several fungal taxa were depleted in patients with severe/critical COVID-19.The distinct compositional changes of the fungal gut microbiome in SARS-CoV-2 infection, especially in severe COVID-19 illness, illuminate the necessity of a broader approach to investigate whether the differences in the fungal gut microbiome are consequences of SARS-CoV-2 infection or a predisposing factor for critical illness.

## Introduction

The gut microbiome is a major player in immunomodulation and immune response both locally and in distant tissues (Rhee et al., 2009; Szabo, 2015; Scheffold and Bacher, 2020), up to fecal transplants determining the survival of influenza-infected mice (Zhang et al., 2020). Metabolites like butyrate are heavily implicated in immunomodulation (Chriett et al., 2019), circulate systemically (Ritzhaupt et al., 1998) and influence the severity of lung infections (Haak et al., 2018; Chemudupati et al., 2020).

Besides the bacterial kingdom, the microbiome includes viruses, archaea and fungi. These players exist in a complex ecosystem that is likely greater than the sum of its parts, though little is known about its order as of yet. Interaction and interdependence of microorganisms are commonly found in nature, so much that sociomicrobiology is extensively studied beyond medical approaches (Simonet and McNally, 2021). Both crosstalk and inter-kingdom interactions are relevant in the human microbiome, especially in the gut (Kruger et al., 2019). While bacteria, the predominant kingdom, keep fungal growth under control, fungi modify bacterial growth in return as fungi have the capacity for immunomodulation and can therefore aggravate or ameliorate illnesses and affect host reaction to bacteria (van Tilburg Bernardes et al., 2020).

We and others recently demonstrated that the gut microbiome of SARS-CoV-2 infected patients is characterized by a dysbiosis with a reduced bacterial richness, an increased abundance of *Enterobacteriaceae* and depletion of immunomodulatory bacteria (Gu et al., 2020; Zuo et al., 2020b; Reinold et al., 2021; Yeoh et al., 2021). Several bacterial producers of butyrate, a short-chain fatty acid known for anti-inflammatory effects (Segain et al., 2000), are demonstrably reduced in COVID-19 illness (Tang et al., 2020; Moreira-Rosario et al., 2021; Reinold et al., 2021).

Considering that severe cases of COVID-19 involve a dysregulated immune response with a lower abundance of T killer cells and a cytokine storm (Coperchini et al., 2021) and fungi are known to shape immunological responses and T cell action (Enaud et al., 2018; Scheffold et al., 2020), it is astounding that the fungal microbiome in COVID-19 has been largely overlooked so far. Just few studies have investigated the fungal gut microbiome in SARS-CoV-2 infected patients to date, one including 30 hospitalized COVID-19 patients (Zuo et al., 2020a) and the other analyzing 67 COVID-19 patients (Lv et al., 2021).

Our study aims to characterize the composition of the fungal microbiome linked to SARS-CoV-2 infection and COVID-19 severity in a cohort of 53 patients in order to assess the impact of fungal dysbiosis in the context of this pandemic. We found major differences in alpha and beta diversity in fungal blooms between mild and severe cases of COVID-19 and distinct differences in the interconnected fungal communities between SARS-CoV-2 positive and SARS-CoV-2 negative patients, raising the question of the role of the fungal microbiome in shaping the course of COVID-19.

## Material and Methods

### Study Population

The Ethics Committee of the Medical Faculty of the University of Duisburg-Essen reviewed and approved this study (20-9237-BO) which was performed in accordance with the latest version of the Declaration of Helsinki. Written informed consent was obtained from all patients before enrolment. All samples included in this study were part of a cohort of 212 patients presenting in the tertiary care University Hospital Essen between April and November 2020 that comprised 117 SARS-CoV-2 positive and 95 SARS-CoV-2 negative patients with other reasons for hospital presentation described elsewhere (Reinold et al., 2021). SARS-CoV-2 real-time PCR from nasopharyngeal swabs was used for detection of acute SARS-CoV-2 infection in all patients enrolled in this study. Pneumonia was diagnosed by low-dose computer tomography or chest x-ray. COVID-19 severity illness was classified according to the WHO (Agarwal et al., 2020). Non-severe COVID-19 was defined as SARS-CoV-2 infection without pneumonia or pneumonia with a saturation of peripheral oxygen (SpO2) > 90% on room air and typical signs such as fever and cough. Severe COVID-19 was defined as symptomatic pneumonia with SpO2 < 90% on room air and a respiratory rate > 30 breaths per minute or clinical signs of severe dyspnoea. Critical disease was classified by the need of life-sustaining treatment, e.g. acute respiratory distress syndrome or septic shock. Severe and critical COVID-19 patients were assigned to one category (severe/critical) because of the low number of patients with critical COVID-19. Initially, the ITS2 region was amplified in all rectal swab samples of the 212 patient samples of the larger cohort. Only the 53 patients who exhibited visible ITS2-region amplicons in agarose gel electrophoresis after 32 rounds of PCR-amplification were included in this study and their amplicons were used for ITS-2 sequencing. Of these 53 patients, 30 patients were SARS-CoV-2 positive, including 21 patients with non-severe COVID-19 and 9 severe/critical COVID-19 illness.

### Sample Processing

Samples of SARS-CoV-2 positive and negative individuals were processed simultaneously, including DNA extraction, amplicon PCRs, library preparation and amplicon sequencing. Rectal swab samples from the DNA/RNA Shield™ Collection Tube with Swab (Zymo Research, Freiburg, Germany) were used and stored in stabilizing DNA/RNA shield at -80°C until DNA extraction was performed. DNA was extracted using the ZymoBIOMICS DNA Miniprep Kit (Zymo Research) with a bead-beating step with the Fast-Prep device (MP Biomedicals, Santa Ana, CA) prior to DNA extraction. Libraries were prepared using the NEBNext^®^ Ultra II DNA Library Prep Kit. The ITS2 region was amplified with ITS3 (5′-GCATCGATGAAGAACGCAGC-3′) and ITS4 (5′-TCCTCCGCTTATTGATATGC-3′) primers and samples were sequenced on a NovaSeq 6000 SP flowcell with PE250.

### Microbiome Data Analysis

Demultiplexed fastq-files of forward and reverse reads per sample were analyzed using the QIIME2 (Quantitative Insights Into Microbial Ecology2) pipeline (Bolyen et al., 2019) with the DADA2-package. Chimeric sequences were filtered using the consensus method. We obtained a total of 3,972,700 quality-filtered sequences of 53 samples with a minimum of 15,821 and maximum of 128,935 sequences per sample and a mean/median number of 74,957/78,300 sequences per sample. Alpha diversity and beta diversity metrics were calculated with a rarefaction depth of 15,821 sequences. Taxonomy was assigned with a Naïve Bayes classifier, trained on the UNITE ITS database QIIME2 release (Version 8). Alpha diversity metrics, barplots, boxplots and Principal Coordinates Analysis (PCoA) were visualized with Dokdo, Version 1.7. Comparisons between categorical metadata columns and alpha diversity metrics were computed with Kruskal-Wallis test. To test for significant differences in beta diversity among groups, we used permutational multivariate analysis of variance (PERMANOVA) of distance matrices with 999 permutations. Biomarkers were assessed using LEfSe (Linear discriminant effect size analysis) (Segata et al., 2011) with p<.05 for fungal class comparison using Kruskal-Wallis test and a linear discriminant analysis (LDA) score >3.5. Taxa that could not be assigned to the phylum level were excluded from biomarker analysis and co-occurrence analysis.

Following the protocol described previously (Kehrmann et al., 2019), the co-occurrence analysis was performed on genus level for fungi. To remove the effect of unbalanced sample sizes in the positive and negative groups, the minimum acceptable threshold was calculated by iterative sub-sampling inside the groups with the smaller group size (N=23). The genus level was used for the comparison of the fungal co-occurrence networks and genera with less than 4 co-occurrences were ignored. The networks were compared and visualized with CompNet, Linux 64-bit version (Kuntal et al., 2016). Closely connected communities in the union network were automatically detected by CompNet. CompNet uses the force-directed layout (from R igraph library) for graph representation by default and implements ‘fastgreedy.community’ (from R igraph library) for community detection.

For the inter-kingdom co-occurrence analyses we also used the bacterial biom-table generated from *16S rRNA* sequencing data of our previous work (Reinold et al., 2021). Samples were filtered so that the resulting *16S rRNA* biom-table contained only those 53 patient samples with available *ITS2*-region sequencing data. Bacterial and inter-kingdom co-occurrence analysis was performed in an analogous manner as the fungal co-occurrence analysis.

Patient characteristics were tested using t-test for metric variables and Fisher exact test for categorical variables with a significance level <.05 using SPSS software (version 27.0).

## Results

### Clinical Characteristics of the Study Population

Of 53 patients included for ITS2-sequencing, 23 patients were SARS-CoV-2 negative and 30 SARS-CoV-2 positive, including 21 patients with non-severe COVID-19 and 9 with severe/critical illness as classified by the WHO. Only one patient (1.9%) received antibiotics and no patient received antifungal therapy at the time point of rectal swab sampling. Variables that had been associated with alterations of the gut microbiome previously, including ethnicity, body-mass-index (BMI), smoking status and most medication including metformin, proton-pump inhibitors, laxatives and corticosteroids, taken at the time point of sampling did not differ between the groups of SARS-CoV-2 negative patients and patients with non-severe and severe/critical COVID-19 illness (**Table 1**). The period between hospital admission and the time of rectal swab sampling was comparable for the groups. Male sex was more frequent in the group of SARS-CoV-2 negative patients 17 (74%) compared with non-severe (29%) and severe/critical COVID-19 illness (33%, p=.006). The age of patients with severe COVID-19 was higher compared with the other groups (p=.029). Comorbidities did not differ significantly between groups except for hypertension (p=.008), which was less frequently present in patients with non-severe COVID-19 compared to the other two groups. Characteristic symptoms for SARS-CoV-2 infection including cough (p=.017), fever (p=.001) and odor disorders (p=.001) were more prevalent in SARS-CoV-2 infected patients with severe/critical and non-severe COVID-19 illness compared with SARS-CoV-2 negative patients.

**Table 1:** Characteristics of SARS-CoV-2 negative and positive patients linked to COVID-19 disease severity.

Variables	SARS-CoV-2 negative	Non-severe	Severe	P-Value^a^
Number of patients	23	21	9	
Age, years, mean ± SD	59 ± 23	52 ± 19	73 ± 9	.029
Sex, male	17 (74)	6 (29)	3 (33)	.006
Body mass index, kg/m^2^, mean ± SD	30 ± 9	28 ± 5	29 ± 7	.591
Smoker	9 (41)	4 (19)	1 (13)	.229
**Outcome & Complications**				
Length of hospitalization, mean ± SD	7 ± 9	6 ± 8	14 ± 12	.091
Time between hospitalization & rectal swab, Days, mean ± SD	2 ± 4	2 ± 5	1 ± 1	.741
Admission to intensive care unit	0	0	3 (33)	.004
Deceased	1 (4)	0	0	1.000
**Acute symptoms**				
Diarrhea	2 (9)	8 (38)	3 (33)	.040
Odor disorder	0	9 (43)	1 (11)	.001
Fever	3 (13)	10 (48)	7 (78)	.001
Cough	5 (22)	12 (60)	6 (67)	.017
**Comorbidities**				
Pre-existing illness	21 (96)	17 (81)	9 (100)	.186
Diabetes mellitus	8 (35)	3 (14)	4 (44)	.155
Coronary heart disease	6 (26)	1 (5)	2 (22)	.130
Hypertension	15 (68)	5 (24)	6 (67)	.008
Chronic kidney disease	4 (17)	1 (5)	3 (33)	.105
Solid organ transplantation	0	2 (10)	0	.477
Chronic obstructive pulmonary disease	6 (26)	2 (10)	2 (22)	.374
Malignancy	6 (26)	5 (24)	1 (11)	.826
**Medication at timepoint of sampling**				
Proton pump inhibitor	11 (50)	8 (38)	5 (56)	.618
Metformin	3 (13)	0	1 (11)	.216
Laxatives	0	1 (5)	0	.577
ACE inhibitor	13 (62)	3 (14)	6 (67)	.002
Beta Blocker	12 (55)	5 (24)	4 (44)	.106
Platelet aggregation inhibitor	6 (26)	2 (10)	4 (44)	.089
Statin	5 (23)	3 (14)	1 (11)	.708
Steroid	4 (17)	6 (29)	2 (22)	.757
Thyroid hormone substitution	5 (23)	5 (24)	3 (33)	.833
Remdesivir	0	0	1 (11)	.417
Antibiotics	0	0	1 (11)	.170

Data are presented in number (percentage) unless otherwise indicated. Mean ± SD is used for metric variables. ^a^Using One- way ANOVA for metric, Fisher’s exact test and Kruskalli- Wallis test for categorial variables. ACE, angiotensin-converting enzyme; SD, standard deviation.

### Fungal Gut Microbiome of Patients With Severe/Critical COVID-19 Illness Is Characterized by a Reduced Alpha Diversity and Significant Enrichment of the Relative Abundance of Ascomycota Phylum

**Figure 1:**
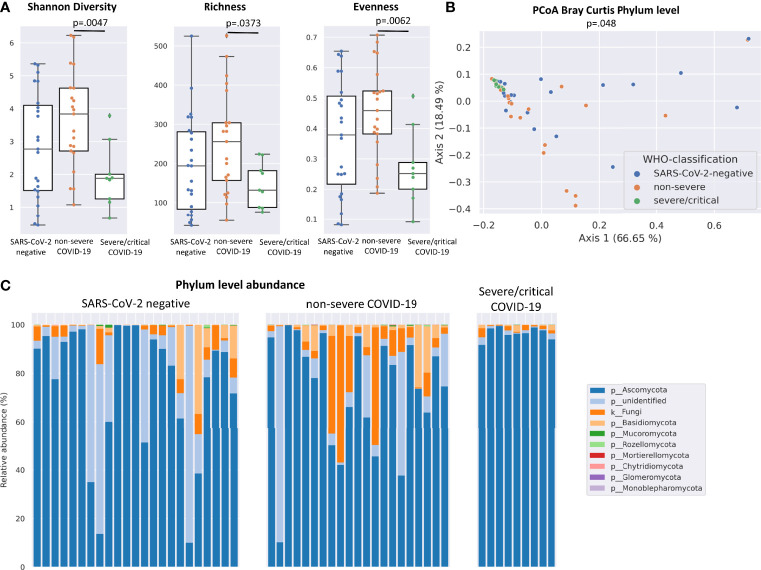
Differences in the fungal gut microbiome in SARS-CoV-2 negative patients and patients with non-severe and severe/critical COVID-19 illness. **(A)** Shannon diversity, richness and evenness of the fungal gut microbiome. Kruskal-Wallis test was used to test for significant differences between groups. **(B)** Principal Coordinates Analysis (PCoA) of phylum level distance matrix. PERMANOVA multivariate analysis was used to test for significant differences between groups. **(C)** Relative phylum level abundance of the fungal gut microbiome in individual SARS-CoV-2 negative patients, patients with non-severe and severe/critical COVID-19.

Significant differences of alpha and beta diversity characterized the fungal gut microbiome of patients with severe/critical COVID-19 illness. Patients with severe/critical COVID-19 illness exhibited a lower Shannon diversity (p=.0047), richness (p=.0373) and evenness (.0062) of the fungal gut microbiome compared with non-severe COVID-19 patients (**Figure 1A**). Principal coordinates analysis (PCoA) of Bray Curtis phylum level distance matrix revealed clustering of patients with severe/critical COVID-19 illness and PERMANOVA multivariate analysis confirmed significant phylum level differences between the three groups (p=.048, **Figure 1B**). The mean relative abundance of the dominating phylum Ascomycota was higher in patients with severe COVID-19 (96.5%) compared with 72.77% in patients with non-severe COVID-19 and 74.6% in SARS-CoV-2 negative patients (**Figure 1C**).

**Figure 2:**
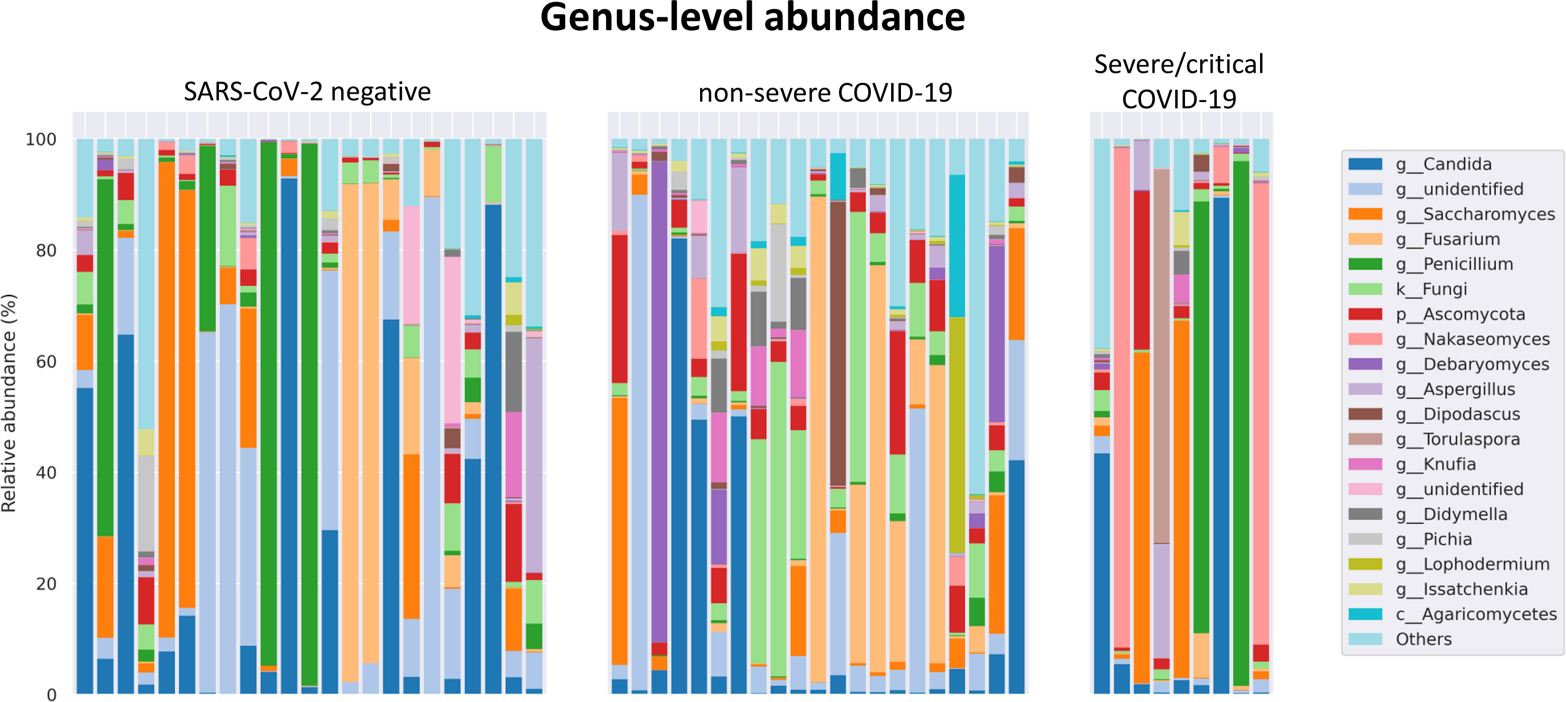
Relative genus level abundance of 20 most abundant genera of the fungal gut microbiome in individual SARS-CoV-2 negative patients, patients with non-severe and severe/critical COVID-19.

**Figure 3:**
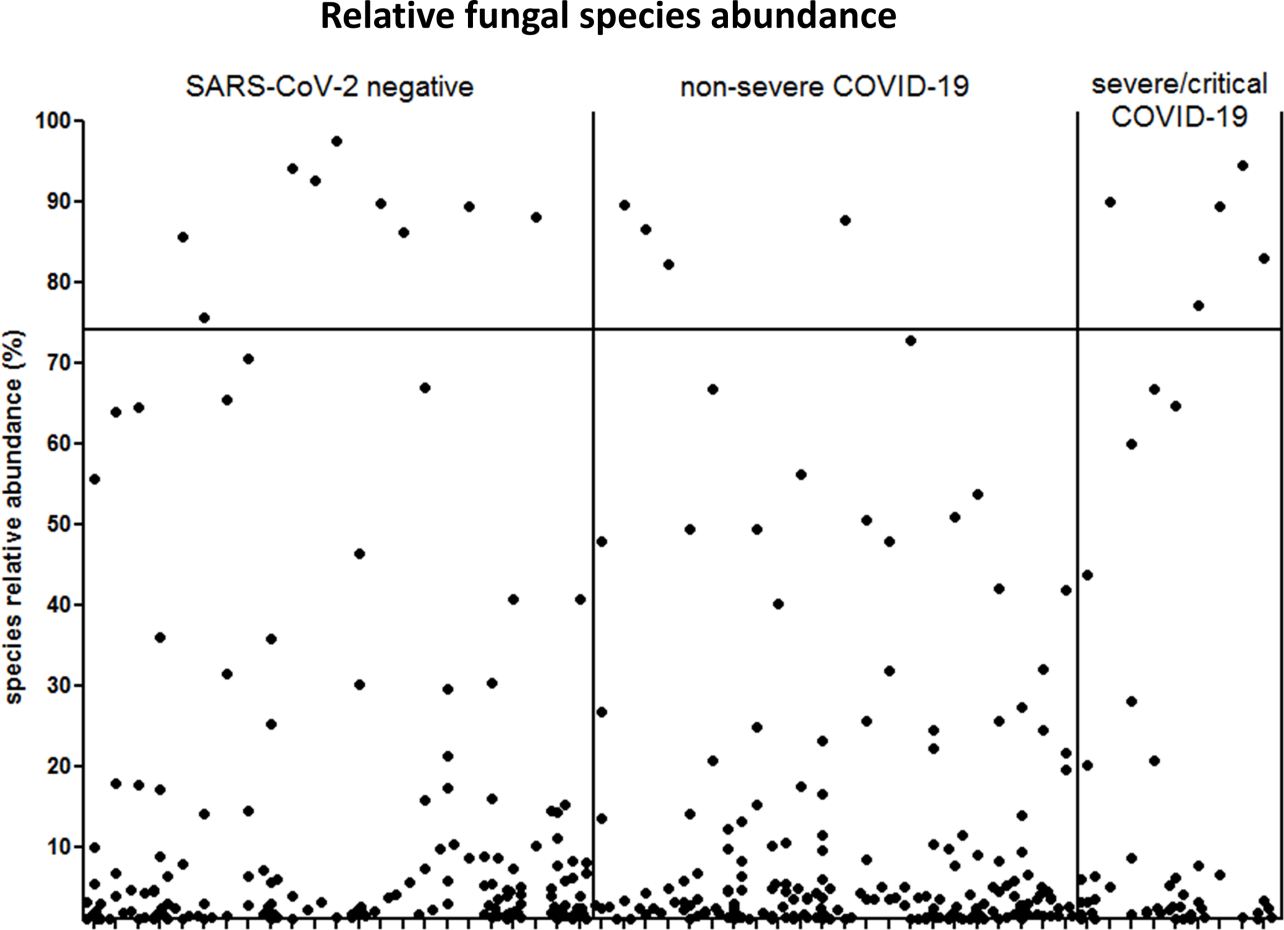
Relative abundance of individual fungal species SARS-CoV-2 negative patients and patients with non-severe and severe/critical COVID-19. Each dot represents one species. The samples of the individual patients are arrayed along the x-axis and the relative abundance of each species is illustrated on the y-axis. Species that account for at least 1% relative abundance are included. The horizontal bar indicates the 75% threshold.

**Figure 4:**
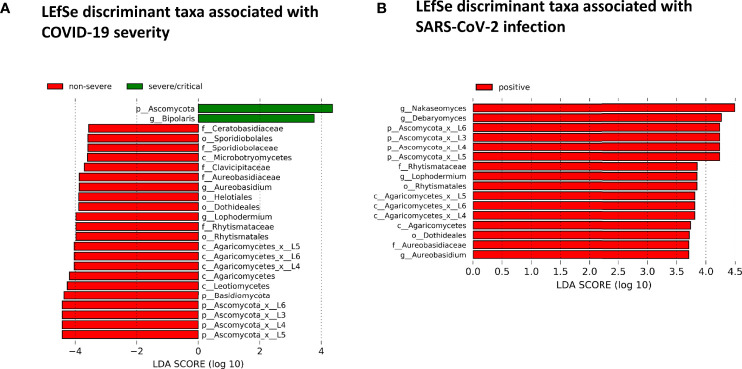
**(A)** Linear discriminant effect size (LEfSe) analysis for identification of fungal taxa enriched in severe/critical COVID19 (green) or non-severe COVID-19 (red). **(B)** LEfSe for identification of fungal taxa enriched in SARS-CoV-2 positive (red) or SARS-CoV-2 negative patients with a minimum linear discriminant abundance value of 3.5 (LDA>3.5).

The fungal gut microbiome was characterized by a high inter-individual dominating genus variation between individual patients also within a group (**Figure 2**). A single species dominated the fungal gut microbiome in 18 of 53 patients with a minimum relative abundance of 75% (**Figure 3**). Patients with severe/critical COVID-19 exhibited a dominating fungal species with a relative abundance of 75% more frequently (5 of 9, 55%) compared with patients with non-severe COVID-19 (4 of 21, 19%) and SARS-CoV-2 negative patients (9 of 23, 39%). The finding of a dominating genus of the gut microbiome is in contrast to the bacterial gut microbiome, that typically does not exhibit a single bacterial genus with a comparable high relative abundance. Linear discriminant analysis of effect size (LEfSe) identified the phylum Ascomycota and the genus *Bipolaris* enriched in severe/critical COVID-19 compared with non-severe COVID-19 illness, while a total of 22 taxa were enriched in non-severe COVID-19 illness including the phylum Basidiomycota and the genera *Lophodermium* and *Aureobasidium* with LDA score >3.5 (**Figure 4A**).

While no taxon was enriched in SARS-CoV-2 negative patients, 16 taxa of the fungal gut microbiome with LDA score >3.5 were enriched in SARS-CoV-2 positive patients when comparing SARS-CoV-2 positive and negative patients. Among these were the yeast *Debaryomyces* and black yeast-like fungus *Aureobasidium* (**Figure 4B**). Twelve of these 16 taxa were taxa of the Ascomycota and four of the Basidiomycota phylum (all of the class Agaricomycetes).

### Pronounced and Distinct Interconnected Fungal Communities Discriminate SARS-CoV-2 Positive and SARS-CoV-2 Negative Patients

**Figure 5:**
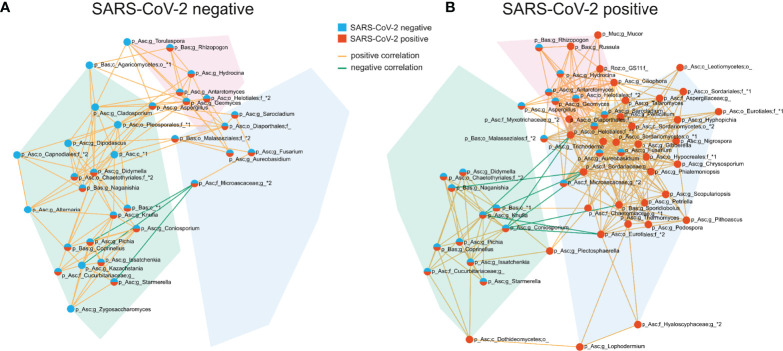
Co-occurrence networks of fungal genera in SARS-CoV-2 negative **(A)** and SARS-CoV-2 positive patients **(B)**. The interconnected communities are visualized by transparent polygons in blue, green and purple. Nodes represent fungal genera and the colour of the nodes visualizes the group association. Genera included in the SARS-CoV-2 negative interconnected community are illustrated in blue, SARS-CoV-2 positive in red and genera included in interconnected communities of both groups are illustrated in blue and red. Genera exhibiting positive correlations are connected by orange lines, genera exhibiting negative correlations are connected by green lines. Each node is labelled with the genus or the highest taxonomic level that could be assigned by UNITE database and with the phylum (p_) the genus is assigned to. The phylum names are abbreviated in the figure: Asc, Ascomycota; Bas, Basidiomycota; Muc, Mucoromycota; Roz, Rozellomycota.

The competition for nutrients or the production of metabolites affecting fungal growth are mechanisms by which fungi directly interact and affect the composition of the fungal gut microbiome (Richard and Sokol, 2019). To gain a better understanding of these networks, we analyzed the correlation at the genus level of the fungal gut microbiome. SARS-CoV-2 negative and SARS-CoV-2 positive patients were both characterized by three interconnected communities with positive co-occurrences between the members of each community. Interestingly, the members of these communities, as well as their interactions, differed significantly between both groups (**Figure 5** and **Supplementary Figure 1**). The genera *Fusarium*, *Gibberella*, *Sarocladium*, *Aureobasidium*, *Geomyces*, *Trichoderma* and *Phialemoniopsis* were among the genera with the highest connectivity of positive correlations. The largest interconnected community (blue transparent polygon of **Figure 5**) was present in SARS-CoV-2 positive patients consisting of 32 positively-correlated genera with 30 genera of the Ascomycota and two genera of the Basidiomycota phylum. It comprised the genera *Aureobasidium* and *Lophodermium*, which are overrepresented in SARS-CoV-2 positive patients. Fungal species of the genus *Aureobasidium* produce antimicrobial peptides, as do other fungi in this interconnected community like *Fusarium* (Antonissen et al., 2014), *Penicillium*, and *Trichoderma* (Stracquadanio et al., 2020). This fungal sub-network comprised only 5 genera in SARS-CoV-2 negative patients. A second interconnected community (green) was more developed and made up of 20 fungal genera in SARS-CoV-2 negative patients compared with SARS-CoV-2 positive patients (13 genera). The only genera assigned to other phyla than Ascomycota and Basidiomycota that were present in interconnected communities of the co-occurrence networks were the genera *Mucor* of Mucoromycota and a genus of Rozellomycota. Both were members of the third interconnected community in SARS-CoV-2 positive patients (purple) containing 9 genera. They were both uncorrelated to the 6 genera of this interconnected network in SARS-CoV-2 negative patients. Overall, this analysis shows a strong modulation of positive associations between fungal genera in SARS-CoV-2 positive in comparison to SARS-CoV-2 negative patients.

**Figure 6:**
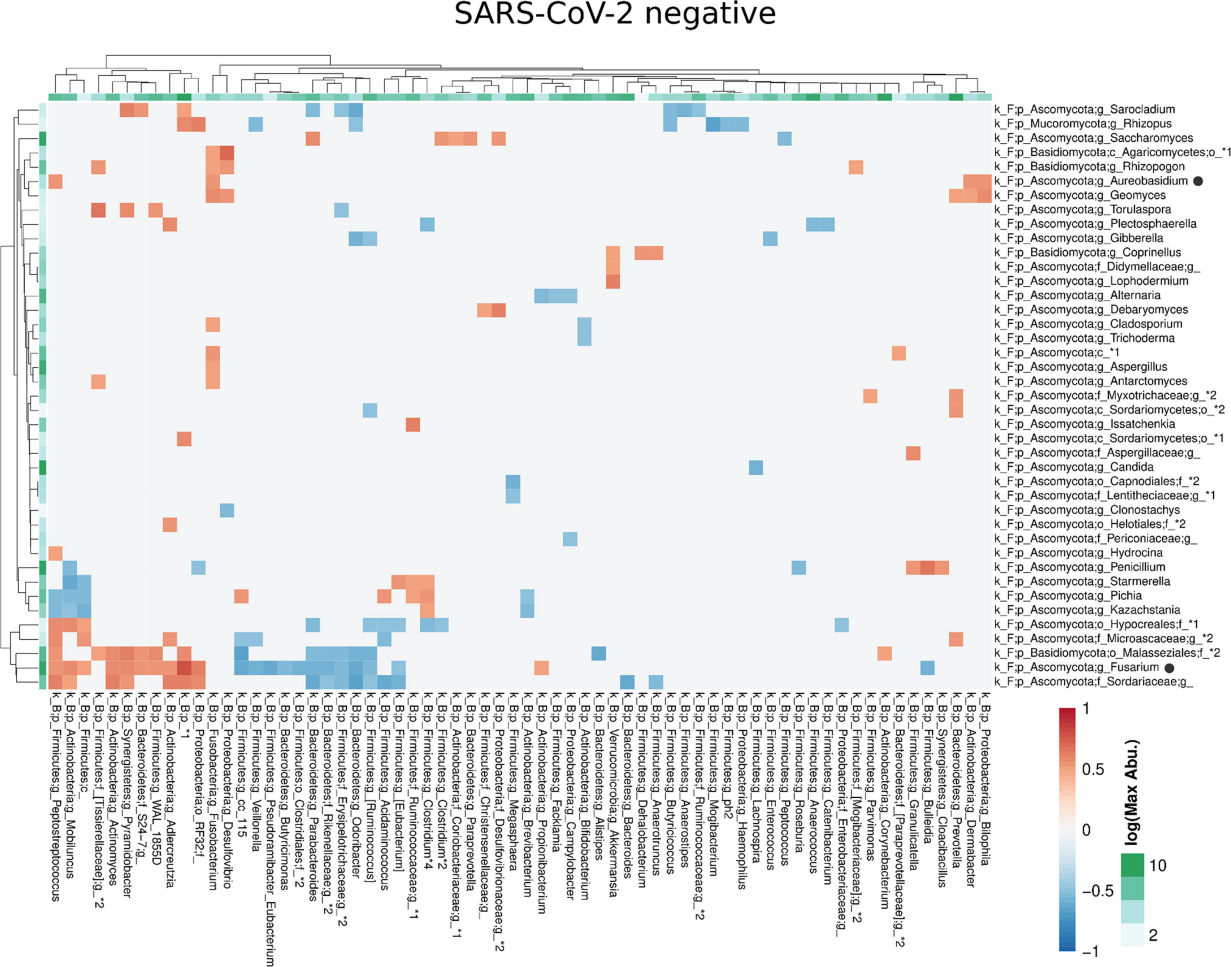
Co-occurrence matrix visualizing the co-occurring genera between the fungal and bacterial kingdoms that exhibit positive (red) or negative (blue) correlations in SARS-CoV-2 negative patients. The colours of the scale bar denote the strength of the correlation with 1 indicating a perfect positive and -1 a strong negative correlation between the two co-occurring genera. The sidebars show the logarithm of the maximum abundance of the genera in the group. When two genera with the same taxonomy assignment were present that could only be identified to the family level or above this level, an asterixis and an identifier number was added to the name to distinguish the taxa. Black circles following the genus names indicate the genera which are discussed in the manuscript.

**Figure 7:**
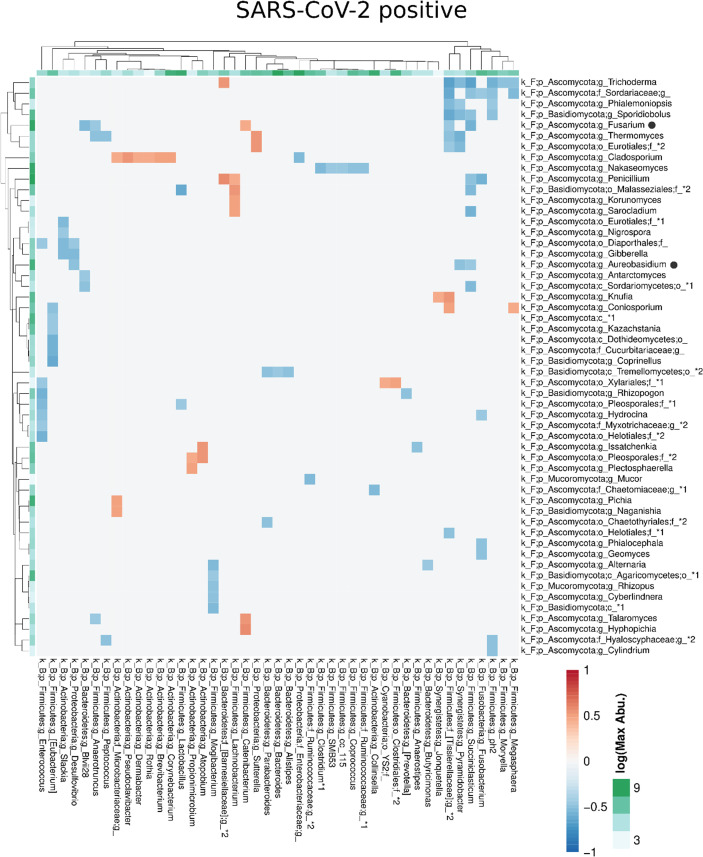
Co-occurrence matrix visualizing the co-occurring genera between the fungal and bacterial kingdoms that exhibit positive (red) or negative (blue) correlations in SARS-CoV-2 positive patients. The colours of the scale bar denote the strength of the correlation with 1 indicating a perfect positive and -1 a strong negative correlation between the two co-occurring genera. The sidebars show the logarithm of the maximum abundance of the genera in the group. When two genera with the same taxonomy assignment were present that could only be identified to the family level or above this level, an asterixis and an identifier number was added to the name to distinguish the taxa. Black circles following the genus names indicate the genera which are discussed in the manuscript.

Compared with the pronounced interconnected networks existing within both, the fungal and bacterial kingdoms in SARS-CoV-2 positive and negative patients, the inter-kingdom correlations between fungi and bacteria were sparse with smaller interconnected communities (**Supplementary Figure 2**). However, distinct arrays of inter-kingdom correlations differentiated the two groups (**Figures 6**, **7**). The gut microbiome of SARS-CoV-2 positive patients showed less positive inter-kingdom correlations and specifically, the largest positively-correlated community of SARS-CoV-2 negative patients, that included *Fusarium*, was not present in SARS-CoV-2 positive patients. Positive correlations of the other, less-pronounced, positively-correlated community in SARS-CoV-2 negative patients, that included *Aureobasidium*, were also largely absent in the SARS-CoV-2 positive patients. The two genera *Bifidobacterium* and *Roseburia* that have anti-inflammatory properties and have been reported to be depleted in SARS-CoV-2 positive patients or patients with severe COVID-19 illness (Moreira-Rosario et al., 2021; Reinold et al., 2021; Yeoh et al., 2021), were not involved in inter-kingdom correlations in SARS-CoV-2 postitive patients but were negatively associated with fungal taxa (*Cladosporium* and *Trichoderma* for *Bifidobacterium* and *Penicillium* for *Roseburia*) in SARS-CoV-2 negative patients.

The fungal gut microbiome between SARS-CoV-2 positive patients with and without diarrhea did not reveal significant differences for alpha and beta diversity metrics. LEfSe did not identify any taxon with LDA >3.5 that discriminated SARS-CoV-2 positive patients with and without diarrhea (data not shown).

## Discussion

Patients with severe/critical COVID-19 illness in our study exhibited a fungal gut microbiome characterized by a reduced Shannon diversity, richness and evenness and by a relative enrichment of Ascomycota compared to patients with non-severe COVID-19 illness. Furthermore, distinct differences in the pronounced interconnected fungal communities characterized the gut microbiome of SARS-CoV-2 positive and negative patients. The interconnected inter-kingdom networks between fungi and bacteria were sparse. Nevertheless, several anti-bacterial metabolites producing fungal genera exhibited different positive and negative correlations with several distinct bacterial genera in both groups, SARS-CoV-2 positive and negative patients.

Major changes of the bacterial gut microbiota have been associated with SARS-CoV-2 infection (Gu et al., 2020; Zuo et al., 2020b; Moreira-Rosario et al., 2021; Reinold et al., 2021; Yeoh et al., 2021; Zuo et al., 2021). In line with our study, the previous studies on the fungal gut microbiome in SARS-CoV-2 infected patients (Zuo et al., 2020a; Lv et al., 2021) also described a dysbiosis of the fungal gut microbiome in SARS-CoV-2 infection. However, the decrease of Shannon diversity and evenness, and the increase of the relative Ascomycota abundance we observed in patients with severe/critical COVID-19 illness, were not reported previously. In line with our study, Lv et al. reported a reduced fungal richness in COVID-19 (Lv et al., 2021). We found a reduced fungal diversity of the gut microbiome in SARS-CoV-2 positive patients, a feature commonly found in patients with inflammatory bowel disease (Chehoud et al., 2015). Lv. et al. (Lv et al., 2021) reported clustering of COVID-19 samples apart from healthy control samples in PCoA, a finding we observed when performing PCoA on phylum level distance matrix. Zuo et al. described a heterogeneous configuration of the fungal gut microbiome during hospitalization of COVID-19 patients (Zuo et al., 2020a), which is in line with our results of the fungal gut microbiome on genus or species level. In our study, severe/critical COVID-19 illness was characterized by depletion of several fungal taxa, a finding reported in COVID-19 patients previously (Lv et al., 2021). The genus *Aspergillus* of the Ascomycota phylum may cause invasive infectious complications in COVID-19 patients (Fekkar et al., 2021). Conflicting data, a depletion of *Aspergillus* (Lv et al., 2021) and an increase of this genus was reported (Zuo et al., 2020a) in the gut microbiome of COVID-19 patients. *Aspergillus* was not significantly associated with COVID-19 severity or SARS-CoV-2 infection in our study. Therefore, fungal genera, which have been reported as producers of antimicrobial metabolites that affect bacterial and fungal growth, including *Debaryomyces* (Banjara et al., 2016) and *Aureobasidium*, were overrepresented in SARS-CoV-2 positive patients of our study. Interestingly, *Debaryomyces* is enriched in the gut microbiome of patients with Crohn´s disease and preferentially inhabits inflamed intestinal tissues (Jain et al., 2021). In a mouse model, *Debaryomyces* impairs wound healing by blocking the myeloid cell specific type 1 interferon – CCL5 axis (Jain et al., 2021).

Compared with the bacterial gut microbiome, the fungal gut microbiome is low in diversity and highly variable between individuals (Nash et al., 2017), which is consistent with our data. A single species dominated the fungal gut microbiome, most overwhelmingly in patients with severe COVID-19 illness.

We found an increase in the relative abundance of the Ascomycota phylum with a mean relative abundance of >96% in patients with severe/critical COVID-19 illness. Ascomycota are critically involved in a complex interplay of not only inflammatory but also anti-inflammatory effects, affecting both local and distant organs (Simonet and McNally, 2021). Mono-colonization of the intestine with the commensal Ascomycota species *Candida albicans* or *Saccharomyces cervisiae* protected mice from severe influenza A virus infection and this effect was mediated by mannans, which are components of the cell walls of these fungi (Jiang et al., 2017).

Few data are available on fungi-fungi interactions in the gut, and to our knowledge, the within-kingdom interactions of the fungal gut microbiome in SARS-CoV-2 infected patients has not been investigated to date. We found distinct pronounced interconnected genus-level communities of the fungal gut microbiome between both groups, suggesting disease-specific correlations in the fungal gut microbiome linked to SARS-CoV-2 infection. The largest positively correlated community, distinctively observed in SARS-CoV-2 positive patients, included several producers of antimicrobial metabolites like *Fusarium* (Antonissen et al., 2014), *Penicillium* and *Aureobasidium* (Takesako et al., 1991). The latter was overrepresented in SARS-CoV-2 positive patients. *Fusarium*, one of the genera exhibiting the highest number of positive correlations with other genera in this community, produces a variety of mycotoxins that alter different intestinal defense mechanisms and aggravate infections (Antonissen et al., 2014). *Gibberella*, another genus with a high degree of positive correlations in this interconnected community is associated with chronic intestinal inflammation (Li et al., 2014). Besides competition between microorganisms of the gut microbiome, many observations indicate that interactions may be mutualistic with advantages for both interacting partners or commensalistic, providing an advantage for the other microorganism without advantage for itself (Richard and Sokol, 2019).

Inflammatory bowel disease is a risk factor for hospitalization in COVID-19 (Creemers et al., 2021) and probiotic fungi have positive effects in inflammatory bowel diseases (Guslandi et al., 2003; Jawhara and Poulain, 2007). Moreover, a crosstalk and interaction between fungi and bacteria exists, with fungi promoting shifts of the bacterial gut microbiome (van Tilburg Bernardes et al., 2020). Compared to the pronounced interconnected fungal communities present in SARS-CoV-2 positive and SARS-CoV-2 negative patients, the inter-kingdom co-occurrences were sparse but differed between both groups. However, investigating the effects of individual microorganisms on the entire human microbiome is complex and may be confounded by many factors, so that *in-vitro* experimental settings or organisms are frequently used as models. Interestingly, exposure to *Debaryomyces*, overrepresented in SARS-CoV-2 positive patients of our study, had the power to alter the bacterial gut microbiome in zebrafish larvae (Siriyappagouder et al., 2018).

A strength of our study is that patient groups did not differ significantly for several factors associated with alterations of the gut microbiome, as only one patient received antibiotics and no patient received antifungal therapy before sampling was performed. Nevertheless, sequencing of different amplicons, in our case *16S rRNA* and *ITS2* region, may cause a bias for the inter-kingdom network analyses. Our study is limited by the number of samples included in the study. Although we initially included 212 samples in PCR for ITS2-amplification, only 53 samples exhibited PCR products in agarose gel that were furthermore included in sequencing analysis. This owes to the fact that fungi comprise only a small portion of the microbial biomass and do not readily overgrow in a healthy gastrointestinal tract (Auchtung et al., 2018; Richard and Sokol, 2019). In addition, we cannot exclude, that other factors not matched between the groups, especially age and sex, which have been reported to be associated with alterations of the fungal gut microbiome (Strati et al., 2016), may contribute to the differences linked to SARS-CoV-2 infection and COVID-19 illness, observed in our study. However, the variables age, gender and comorbidity were not associated with significant differences of the fungal diversity, richness, evenness and relative abundance of Ascomycota within the groups of SARS-CoV-2 negative patients, non-severe and severe/critical COVID-19 patients (**Supplementary Figure 3**). Another limitation is the lacking information about the diet of patients which may also affect the bacterial and fungal gut microbiome (David et al., 2014; Auchtung et al., 2018). An altered in-hospital nutrition and treatment of patients with severe/critical COVID-19 on intensive care units might also have an effect on the fungal gut microbiota. However, rectal samples in patients included in our study were taken shortly after hospital admission and no patient was treated on intensive care unit at the time point of rectal swab sampling.

Fungi have indirect effects on immune development by inducing ecological changes in the bacterial gut microbiome but also affect immune development by promoting infiltration of macrophages into lungs of mice in germ-free mice (van Tilburg Bernardes et al., 2020). They induce strong ecological changes to the gut microbiome and synergistic effects of bacteria and fungi in colonization, as well as increased cytokine levels in animals co-inoculated with both bacteria and fungi. Considering the influence of fungi on the bacterial gut microbiome, on cytokine levels and T cells (Bacher et al., 2019; Shao et al., 2019), the relevance of the fungal gut microbiome needs to be further investigated, especially in diseases like COVID-19, which present with a cytokine storm as a major pathophysiological player.

As our study was cross-sectional, rather than longitudinal, we cannot be certain whether the fungal gut microbiome changes after infection and affects the course of disease, or if the composition of gut mycobiome is a predisposing factor for critical illness.

## Conclusions

The distinct compositional differences of the fungal gut microbiome in SARS-CoV-2 infection, especially in severe COVID-19 illness, studied from samples taken unaffected by the use of an antimicrobial therapy, illuminate the necessity of a broader approach to confirm the differences in the fungal gut microbiome in SARS-CoV-2 infection and to investigate whether the fungal microbiome changes are consequences of SARS-CoV-2 infection or a predisposing factor for critical illness.

## Data Availability Statement 

The datasets presented in this study can be found in online repositories. The names of the repository/repositories and accession number(s) can be found below: SRA NCBI, accession number PRJNA794154.

## Ethics Statement

The studies involving human participants were reviewed and approved by Ethics Committee of the Medical Faculty of the University of Duisburg-Essen (20-9237-BO). The patients/participants provided their written informed consent to participate in this study.

## Author Contributions

JKe and AW conceived the study and raised the funding. JKe, AW, JR, and OW designed the study. JR, LB, JKo, OW, SD, MK, and JB collected study specimens. CF and JKe performed the experiments. JR analyzed the clinical data. JKe and FF analysed the microbiome data. JKe, A-KS and FF wrote the original manuscript and drafted it with substantial contributions from all other authors. All authors contributed to the article and approved the submitted version.

## Funding

This work was supported by the Stiftung Universitätsmedizin Essen under Grant number 20204699115.

## Conflict of Interest

The authors declare that the research was conducted in the absence of any commercial or financial relationships that could be construed as a potential conflict of interest.

## Publisher’s Note

All claims expressed in this article are solely those of the authors and do not necessarily represent those of their affiliated organizations, or those of the publisher, the editors and the reviewers. Any product that may be evaluated in this article, or claim that may be made by its manufacturer, is not guaranteed or endorsed by the publisher.

## Supplementary Material

Supplementary Figure 1 Correlation matrix of fungal genera within the interconnected communities identified by CompNet. Correlations that co-occur within the interconnected communities of SARS-CoV-2 negative patients are illustrated in blue. Correlations that co-occur within the communities of SARS-CoV-2 positive patients are visualized in red and correlations that co-occur in both patient groups are visualized equally with blue and red.

Supplementary Figure 2 Co-occurrence matrix visualizing co-occurrence distributions within the bacterial kingdom, within the fungal kingdom and between the bacterial and fungal kingdoms on the genus level in SARS-CoV-2 negative **(A)** and SARS-CoV-2 positive **(B)** patients. Positive co-occurrence between genera is indicated by red and negative co-occurrence by blue colour of the squares. The colours of the scale bar denote the strength of the correlation with 1 indicating a perfect positive and -1 a strong negative correlation between the two co-occurring genera. Bacterial genera are indicated by the orange and fungal genera by pink colour in the sidebars.

Supplementary Figure 3 Differences in the fungal gut microbiome in SARS-CoV-2 negative patients and patients with non-severe and severe/critical COVID-19 illness for Shannon diversity, richness and evenness of the fungal gut microbiome and subanalysis of differences within the three patients groups linked to age, sex, diarrhea and diabetes. Kruskal-Wallis test was used to test for significant differences between groups. No significant differences were present associated with the variables age, sex, diarrhea and diabetes.

## Abbreviations

BMI, body-mass-index; COVID-19, coronavirus disease 2019; LDA, linear discriminant analysis; LEfSe, Linear discriminant effect size analysis; PCoA, Principal Coordinates Analysis; QIIME2, Quantitative Insights Into Microbial Ecology2; SARS-CoV-2, severe acute respiratory syndrome coronavirus type 2; SpO2, Saturation of peripheral oxygen; WHO, World Health Organization.
