# Supplementary figures and images for: The Fungal Gut Microbiome Exhibits Reduced Diversity and Increased Relative Abundance of Ascomycota in Severe COVID-19 Illness and Distinct Interconnected Communities in SARS-CoV-2 Positive Patients
Source: Front Cell Infect Microbiol. 2022 Apr 19;12:848650. doi: 10.3389/fcimb.2022.848650 (PMC9062042; doi:10.3389/fcimb.2022.848650)

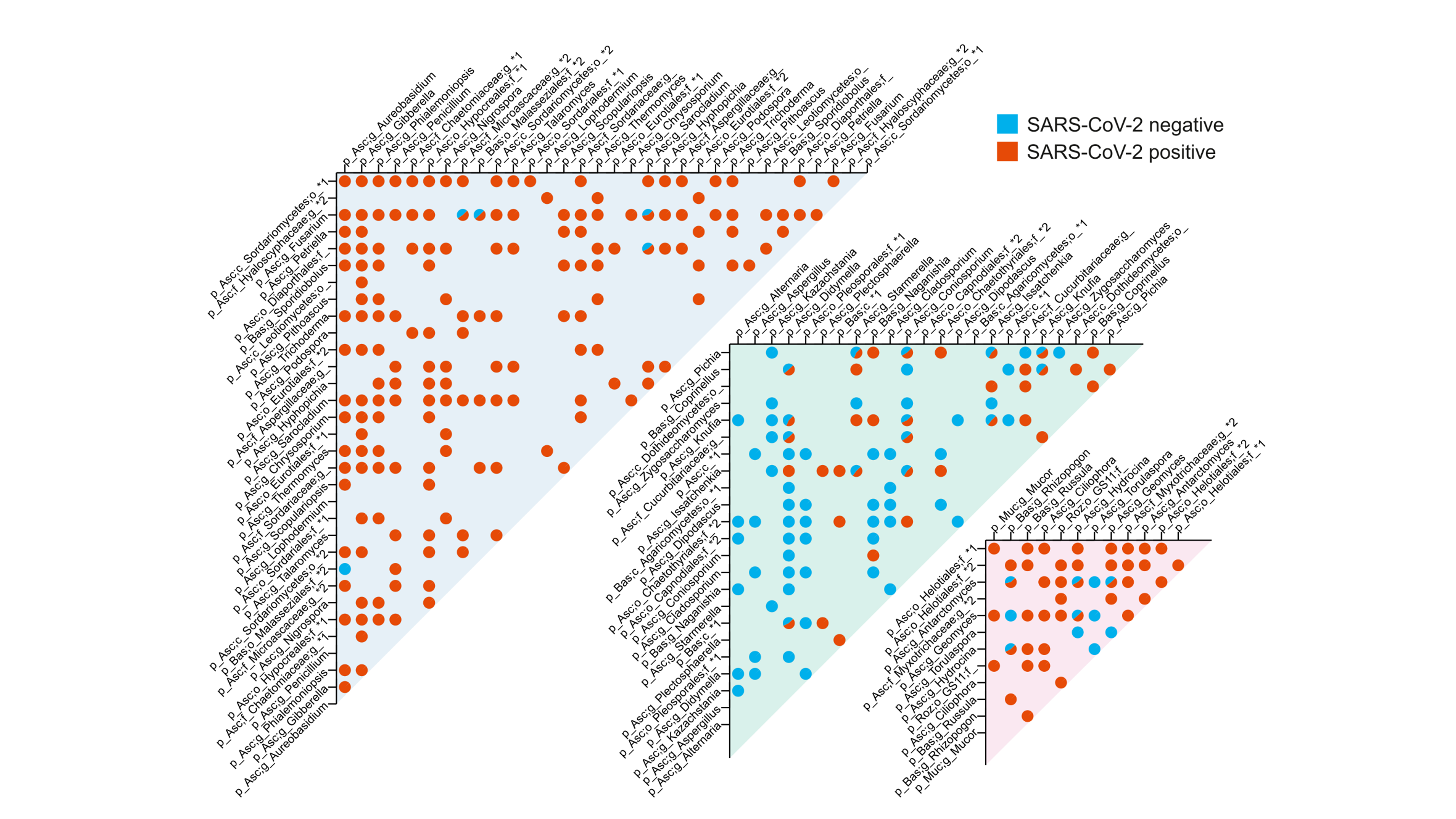

Supplement: Supplementary Figure 1 — Correlation matrix of fungal genera within the interconnected communities identified by CompNet. Correlations that co-occur within the interconnected communities of SARS-CoV-2 negative patients are illustrated in blue. Correlations that co-occur within the communities of SARS-CoV-2 positive patients are visualized in red and correlations that co-occur in both patient groups are visualized equally with blue and red. [file Image_1.tif]

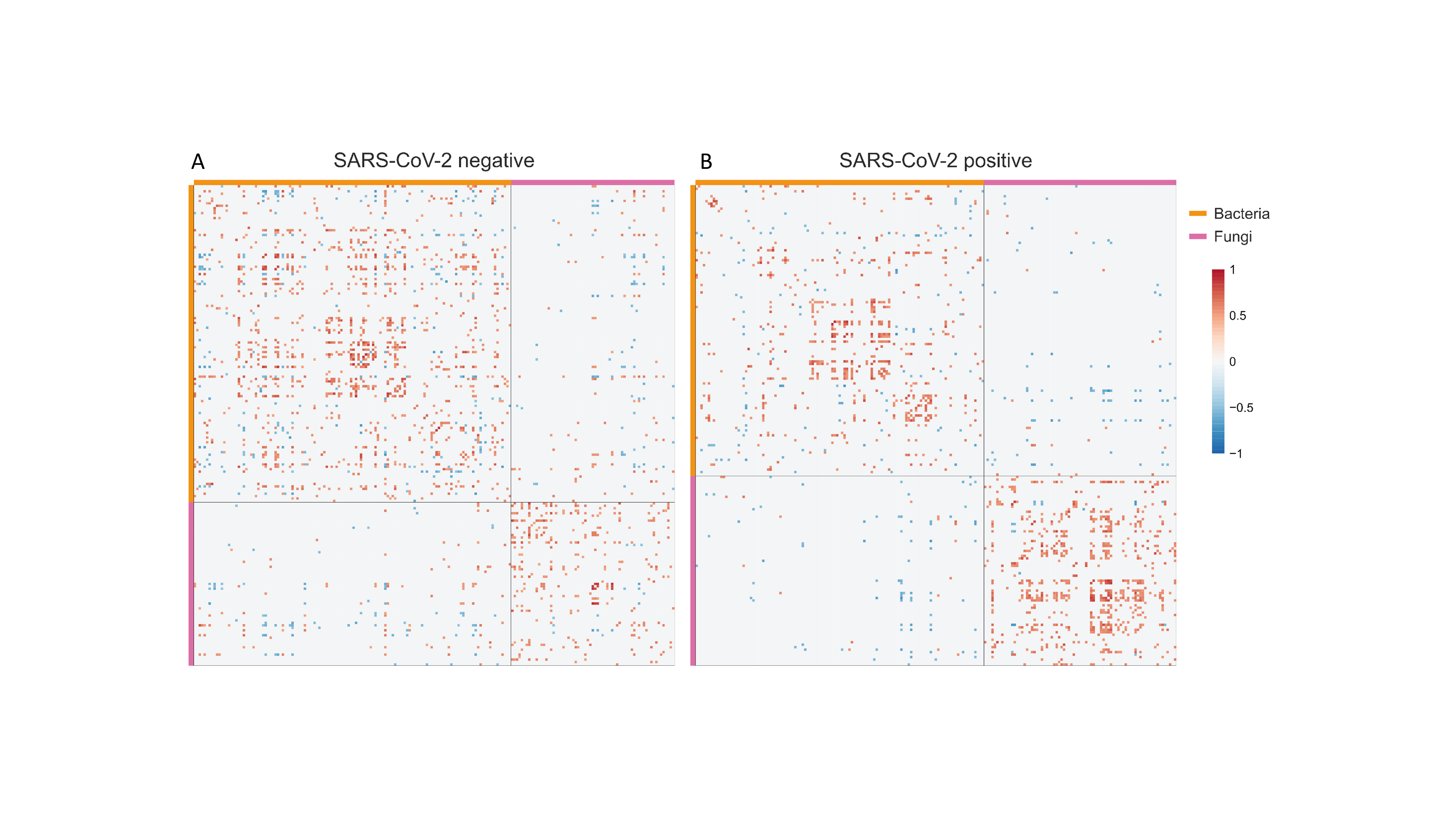

Supplement: Supplementary Figure 2 — Co-occurrence matrix visualizing co-occurrence distributions within the bacterial kingdom, within the fungal kingdom and between the bacterial and fungal kingdoms on the genus level in SARS-CoV-2 negative (A) and SARS-CoV-2 positive (B) patients. Positive co-occurrence between genera is indicated by red and negative co-occurrence by blue colour of the squares. The colours of the scale bar denote the strength of the correlation with 1 indicating a perfect positive and -1 a strong negative correlation between the two co-occurring genera. Bacterial genera are indicated by the orange and fungal genera by pink colour in the sidebars. [file Image_2.tif]

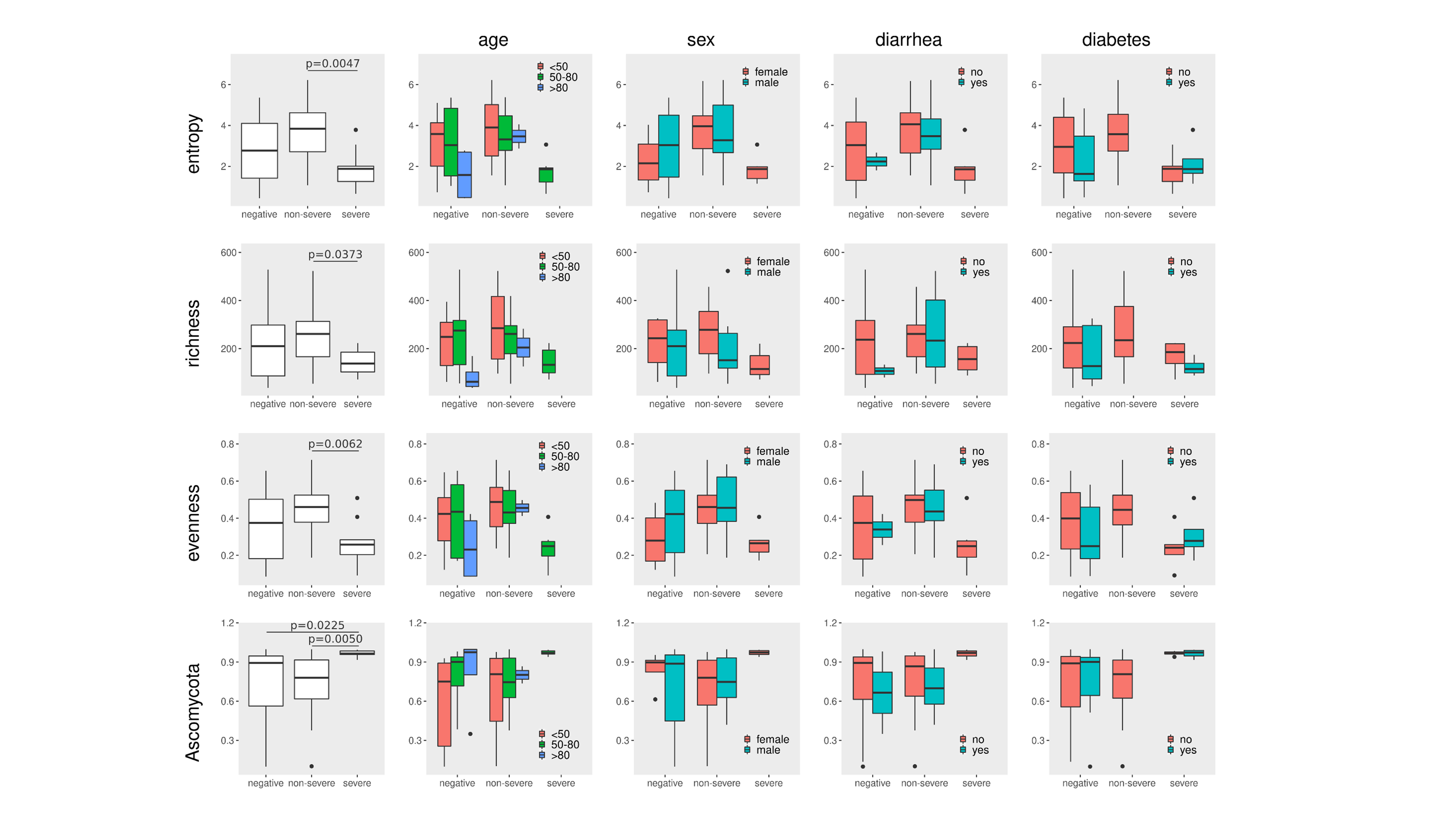

Supplement: Supplementary Figure 3 — Differences in the fungal gut microbiome in SARS-CoV-2 negative patients and patients with non-severe and severe/critical COVID-19 illness for Shannon diversity, richness and evenness of the fungal gut microbiome and subanalysis of differences within the three patients groups linked to age, sex, diarrhea and diabetes. Kruskal-Wallis test was used to test for significant differences between groups. No significant differences were present associated with the variables age, sex, diarrhea and diabetes. [file Image_3.tif]
